# *Spinacia oleracea* L. Baby Leaves as a Source of Bioactive Principles: The Chemical Profiling of Eco-Sustainable Extracts by Using LC-ESI/HRMS- and ^1^H NMR-Based Metabolomics

**DOI:** 10.3390/foods13223699

**Published:** 2024-11-20

**Authors:** Antonietta Cerulli, Luciana Maria Polcaro, Milena Masullo, Sonia Piacente

**Affiliations:** 1 Department of Pharmacy, University of Salerno, Via Giovanni Paolo II 134, 84084 Fisciano, Italy; acerulli@unisa.it (A.C.); lpolcaro@unisa.it (L.M.P.); mmasullo@unisa.it (M.M.); 2 Agritech National Research Center, Corso Umberto 40, 80138 Naples, Italy; 3 PhD Program in Drug Discovery and Development, Università degli Studi di Salerno, Via Giovanni Paolo II 132, 84084 Fisciano, Italy

**Keywords:** baby leaves, *Spinacia oleracea* L., eco-sustainable extractions, ^1^H NMR and HRMS-based metabolomics, 20-hydroxyecdysone, multivariate data analysis

## Abstract

*Spinacia oleracea* L. cultivar platypus leaves are identified as a functional food due to their nutrient composition which promotes health beyond basic nutrition. Considering the increasing use of food supplements, *S. oleracea* baby leaves have been extracted by maceration, solid–liquid dynamic extraction (SLDE)-Naviglio, and ultrasound-assisted extraction (UAE) using EtOH and EtOH:H_2_O mixtures. The analysis of the extracts by using LC-ESI/HRMSMS revealed 42 compounds (flavonoids, polar lipid derivatives, and 20-hydroxyecdysone), along with primary metabolites, detected by NMR analysis. A principal component analysis (PCA) of LC-ESI/HRMS and NMR data was performed, revealing how 20-hydroxyecdysone and flavonoids, the specialized metabolites mainly responsible for the biological activity of *S. oleracea* leaves, occurred in the highest amount in the EtOH and EtOH:H_2_O (70:30, *v*/*v*) extracts obtained by SLDE-Naviglio extraction. 20-hydroxyecdysone was also quantified in all the extracts via LC-ESI/QTrap/MS/MS using the Multiple Reaction Monitoring (MRM) method. The EtOH extracts obtained by SLDE-Naviglio and maceration showed the highest content (82.16 and 81.27 mg/g extract, respectively). The total phenolic content (118.35–206.60 mg GAE/g), the flavonoid content (10.90–41.05 mg rutin/g), and the Trolox Equivalent Antioxidant Capacity (TEAC) (1.63–2.05 mM) of the extracts were determined. The EtOH:H_2_O (70:30, *v*/*v*) extract analyzed by using SLDE-Naviglio showed the highest phenolic and flavonoid content and radical scavenging activity.

## 1. Introduction

Spinach (*Spinacia oleracea* L.) is a leaf vegetable belonging to the Amaranthaceae family. It is an economically important leafy vegetable grown worldwide that is consumed raw, such as in salads, cooked as soup, and steamed [1].

Spinach is widely diffused as a functional food due to its nutritional composition, ascribable to its content of minerals (iron, copper, phosphorous, zinc, selenium), vitamin B complex (niacin and folic acid), ascorbic acid, and omega-3-fatty acids, along with specialized metabolites like flavonoid derivatives and 20-hydroxyecdysone [2,3]. Flavonoids such as spinacetin, patuletin, and methylenedioxyflavone derivatives, previously extracted from spinach leaves via maceration using water or organic solvents [4,5], via pressurized liquid extraction using hexane (70:30), and by using supercritical fluid [6,7], are mainly responsible for the antioxidant properties reported for *S. oleracea* leaves [8,9]. 20-hydroxyecdysone (ecdysterone), previously extracted from spinach leaves via UAE using a mixture of methanol–water (80:20) and natural deep eutectic solvents (NADESs) [10,11] is a functional bioactive compound of *S. oleracea* leaves, reported as the most interesting ingredient of some dietary supplements used mainly by sportspeople to increase physical performance [12,13].

The potential ability of 20-hydroxyecdysone to activate the Mas1 receptor, a key component of the renin–angiotensin system, could explain the diverse pleiotropic effects observed in various animals. The renin–angiotensin system plays a crucial role in regulating blood pressure, fluid balance, and electrolyte homeostasis, but it also has broader physiological implications beyond its traditional cardiovascular functions. In detail, activation of the Mas1 receptor by 20-hydroxyecdysone might promote muscle growth and protein synthesis, potentially through mechanisms involving enhanced nutrient uptake and utilization by muscle cells [14].

In Italy, conventional spinach production increased to 96,860 tonnes in 2022 [15]. During food industrial processes, only leaves are used, while cotyledons, also known as baby leaves, have no commercial value, and consequently, they are considered a waste. A total of 6.5% of fresh spinach is estimated to be lost as waste in the packaging and retail phase of the life cycle [16]. Currently, baby leaves have been little studied from a phytochemical perspective; in fact, there are only few papers in the literature that highlight the presence of flavonoids, previously reported in spinach leaves, with antioxidant activity [17].

Based on these considerations and the interesting biological activity reported for specialized metabolites of *S. oleracea* cultivar platypus, in particular for 20-hydroxyecdysone, growing attention is paid to the possible exploitation and reuse of baby leaves.

With this aim, EtOH and EtOH:H_2_O (80:20, 70:30, 50:50, *v*/*v*) extracts have been obtained by using a conventional extraction procedure (maceration at room temperature) and unconventional extraction methods (ultrasound-assisted extraction (UAE) and solid–liquid dynamic extraction (SLDE-Naviglio)). Herein, for the first time, the extraction of spinach baby leaves by using UAE and SLDE-Naviglio (NAV) was performed. In detail, UAE represents one of the most common, fast, and simple unconventional extraction methods [18]; regarding SLDE-Naviglio, it is considered in line with the green principles and among the green extraction techniques used to improve the sensitivity and the selectivity of analytical methods, ensuring a wide range of advantages such as exhaustion in a short time, with solid matrices containing extractable substances at low operating temperatures, reproducibility of the extraction with a guarantee of the production of high-quality extracts, easy use, low energy consumption, and a fast extraction process [19].

The influence of extraction conditions on the chemical profile of *S. oleracea* baby leaves has been investigated by using a metabolomic approach based on LC-ESI/HRMS and ^1^H NMR analysis of extracts. In this way, primary (amino acids and carbohydrates) and specialized metabolites have been identified. To unambiguously confirm the compounds identified by LC-ESI/HRMS analysis, a chemical investigation of EtOH:H_2_O (70:30, *v*/*v*) extract obtained by using SLDE-Naviglio has been carried out. 20-hydroxyecdysone and the main flavonoids have been isolated, and their structures have been assigned by 1D and 2D-NMR, in combination with flow injection analysis coupled with mass spectrometry (FIA-MS). The impact of the different extraction methods on the chemical profiles of the extracts has been investigated by multivariate data analysis of the LC-ESI/HRMS and ^1^H NMR data. Considering the pharmacological activities reported for 20-hydroxyecdysone [14], it was quantified in all of the extracts by LC-ESI/QTrap/MS/MS using the Multiple Reaction Monitoring (MRM) method, a robust approach for assessing the presence and concentration of this bioactive compound. Finally, the evaluation of the total phenolic and flavonoid content, as well as the radical scavenging activity of *S. oleracea* baby leaf extracts, has been performed, providing valuable insights into their antioxidant potential.

## 2. Materials and Methods

### 2.1. Extraction Workflow

After harvesting, the first leaves of *S. oleracea* L. cultivar platypus were crushed and immediately stored in a freezer at a temperature of −20 °C. After 10 days, they were submitted to freeze-drying. The extracts were prepared using EtOH (Sigma-Aldrich, Milan, Italy) and EtOH:H_2_O (80:20, 70:30, 50:50, *v*/*v*) as extraction solvents. A conventional extraction procedure, such as maceration at room temperature, and two other unconventional procedures, such as UAE and SLDE-Naviglio, were performed. For each extract, the same ratio of dry baby leaves–solvent was used; moreover, each extraction procedure was repeated three times to obtain one final extract for each kind of extraction.

For maceration, 3 g of dried *S. oleracea* baby leaves were extracted with 120 mL of solvent mixtures and stored at room temperature for 3 days; this was repeated three times. After filtration, the solvent was evaporated by using a rotavapor. In this way, 0.11 g, 0.15 g, 0.23 g, and 0.34 g of EtOH, EtOH:H_2_O (80:20, *v*/*v*), EtOH:H_2_O (70:30, *v*/*v*), and EtOH:H_2_O (50:50, *v*/*v*) dry extract/g of dried baby leaves were obtained, respectively.

For UAE, 3 g of dried *S. oleracea* baby leaves were extracted with 120 mL of solvent mixture for 15 min, working with 200 W of power and 44 kHz of frequency. The extractions were repeated three times, and after evaporation of the solvent, 0.06 g, 0.21 g, 0.23 g, and 0.29 g of EtOH, EtOH:H_2_O (80:20, *v*/*v*), EtOH:H_2_O (70:30, *v*/*v*), and EtOH:H_2_O (50:50, *v*/*v*) dry extract/g of dried baby leaves were obtained, respectively.

For SLDE-Naviglio, 15 g of dried *S. oleracea* baby leaves were extracted with 600 mL of solvent mixture following an extractive protocol of 20 extractive cycles of 12 min (9 min in the static phase and 3 min in the dynamic phase). This procedure was repeated three times. Successively, after evaporation of the solvent, 0.05 g, 0.19 g, 0.16 g, and 0.19 g of EtOH, EtOH:H_2_O (80:20, *v*/*v*), EtOH:H_2_O (70:30, *v*/*v*), and EtOH:H_2_O (50:50, *v*/*v*) dry extract/g of dried baby leaves were obtained, respectively.

### 2.2. LC-ESI/HRMSMS Analysis and Multivariate Data Analysis of Specialized Metabolites

*S. oleracea* extracts were analyzed by LC-ESI/HRMSMS (negative ion mode). A Kinetex EVO 5.0 µm column (150 mm × 2.1 mm) (Phenomenex, Aschaffenburg, Germany), and a binary solvent system were employed. ESI source parameters were set in a specific way (Supplementary Materials); Xcalibur version 2.2 software (Thermo Fisher Scientific, Waltham, MA, USA) was used for data acquisition and analysis (Supplementary Materials). After acquisition, in triplicate, the data were filtered using MZ mine 2.38; the resulting csv. file was processed by SIMCA P+ 12.0. For PCA, UV scaling was used to build a matrix composed of 42 variables, corresponding to the peak area of the *m*/*z* values of specific specialized metabolites identified in the LC-ESI/HRMSMS analysis of *S. oleracea* extracts and the 12 observations, represented by the different extracts (Supplementary Materials).

### 2.3. Isolation Procedures

The EtOH:H_2_O (70:30, *v*/*v*) extract obtained by using SLDE-Naviglio has been purified by HPLC-UV (330 nm) and HPLC-RI to afford compounds **6**, **14**, **18**–**20**, trigonelline, and tryptophan (Supplementary Materials). The structures of the isolated compounds were characterized using one-dimensional (1D) and two-dimensional (2D) Nuclear Magnetic Resonance (NMR) analysis.

### 2.4. H-NMR Analysis and Multivariate Data Analysis of Primary Metabolites

NMR analyses were carried out on a Bruker Ascend-600 spectrometer (Bruker BioSpin GmBH, Rheinstetten, Germany); methanol-*d*_4_ (99.95%, Sigma-Aldrich, Milan, Italy) was used as the solvent for pure compounds. After acquisition, the spectra were analyzed using Topspin 3.2 software.

For the targeted multivariate analysis, all extracts were acquired in D_2_O. In the first phase, ^1^H-NMR experiments were performed in triplicate for all of the extracts to assign a key signal to each metabolite. For ^1^H-NMR, phosphate buffer and NaN_3_ were added to each extract to prevent microbial contamination. Subsequently, 13 μL of 3-(trimethylsilyl)-propionic-2,2,3,3-*d*_4_ acid sodium salt (TSP), at 0.9% (*w*/*w*) in D_2_O 99.9 atom %, used as the internal standard, was added to 537 μL of a specific dilution of each extract, at a final concentration of 1 mM. TSP was selected as a reference for the calibration of NMR spectra and as a reference in the quantification of metabolites [20]. The ^1^H NMR spectra were imported into the Processor module of Chenomx NMR Suite version 10.0 (Chenomx, Edmonton, AB, Canada) (Supplementary Materials), where they were subjected to baseline correction, line broadening, phase correction, and shim correction. In this module, the spectra were calibrated to the signal of the internal standard (TSP) and the pH was entered within a certain range (pH 4–9). The Profiler module was used to determine the relative metabolite abundance. A list of compounds and their relative abundance was produced from each spectrum and was subjected to statistical analysis for a given sample (Table S1).

### 2.5. Quantitative Analysis of 20-Hydroxyecdysone *(**6**)*

Quantitative analysis of compound **6** was performed on an LC-ESI/QTrap/MS/MS system (negative ion mode) working in Multiple Reaction Monitoring (MRM) mode [21]. For 20-hydroxyecdysone (**6**) (external standard, ES), all parameters were optimized, and six different ES concentrations (0.1, 1.0, 5.0, 10.0, 20.0, 40.0 µg/mL) were used for the calibration curve. For the *S. oleracea* eco-sustainable extracts, solutions of 0.5 mg/mL (injection volume 5 µL) were prepared and injected in triplicate. The chromatographic method was evaluated for calibration curve linearity, intra- and inter-day precision, and accuracy; the limit of detection (LOD) and quantification (LOQ) were calculated as 0.05 ng/mL and 0.16 ng/mL, respectively.

### 2.6. Determination of Total Phenolic Content, Flavonoid Content, and Radical Scavenging Activity by Spectrophotometric Assays

The total phenolic content, determined using the Folin–Ciocalteu method, and flavonoid content of the extracts were determined as previously reported [22]. The radical scavenging activity of extracts was assessed using a TEAC assay (Supplementary Materials).

## 3. Results and Discussion

### 3.1. Fingerprint of Extracts of S. oleracea by LC-ESI/HRMSMS

With the aim of evaluating the possible use of *S. oleracea* baby leaves as a nutraceutical product, different fast and simple extractions were performed. Herein, EtOH and EtOH:H_2_O (80:20, 70:30, 50:50, *v*/*v*) were selected as solvent mixtures. Since the extraction procedures can significantly influence the kind of metabolites present in the extracts, maceration (MAC) at room temperature, considered a conventional procedure, and UAE and SLDE-Naviglio (NAV) as unconventional procedures were selected. In order to obtain a preliminary profile of metabolites in the eco-sustainable extracts of *S. oleracea*, an analytical approach based on LC-ESI/HRMSMS analysis was carried out.

**Figure 1:**
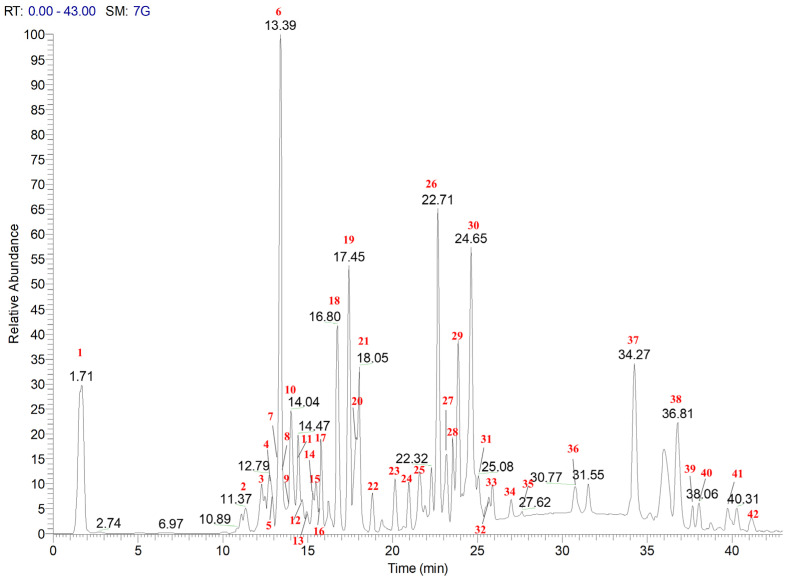
LC-ESI/HRMS profile of baby leaves of *S. oleracea* EtOH (100%) extract obtained by using SLDE-Naviglio. Legend: **1**: allantoin; **2**–**5** and **7**–**20**: flavonoids; **6**: 20-hydroxyecdisone; **23**: triterpene saponin; **21**, **22**, **31**, and **33**: oxylipins; **25**, **28**, **29**, and **32**: sucrose-monoacylglycerol (SCMG); **26**, **27**, **30**, and **34**–**42**: glycolipids; **24**: trymethylellagic acid.

In Figure 1, the LC-ESI/HRMS profile of baby leaves of *S. oleracea* EtOH extract obtained by using SLDE-Naviglio is reported; the peaks corresponding to 42 metabolites could be observed. In detail, the careful study of the exact mass, fragmentation pattern, and literature data prompted us to tentatively identify allantoin (**1**), flavonoids (**2**–**5**, **7**–**20**), steroid (**6**), triterpene saponin (**23**), oxylipins (**21**, **22**, **31**, **33**), sucrose-monoacylglycerol (SCMG) (**25**, **28**, **29**, **32**), glycolipids (**26**, **27**, **30**, **34**–**42**), and trymethylellagic acid (**24**) (Table 1).

The highest peak in the LC-ESI/HRMS profile showed a pseudomolecular ion at *m*/*z* 525.3069 (**6**) attributable to a [(M+FA)-H]^−^ formic acid adduct of C_27_H_44_O_7_; through careful analysis of the MS/MS spectrum and literature data, compound **6** was assigned as 20-hydroxyecdysone, a polyhydroxylated steroid derivative that retains the full C8 sterol side-chain, with a 14-hydroxy-7-en-6-one chromophoric group located in the B-ring and an A/B-*cis*-ring junction. It is considered a marker compound of *S. oleracea* leaves [23].

From the LC-ESI/HRMSMS spectra, compounds **2**–**5**, **7**–**17**, and **18**–**20** were assigned as flavonoid derivatives. The analysis of fragmentation spectra gave us information about the kind of flavonoid units and the type of sugar unit, as well as the presence of esterification with phenylpropanoid moieties. In detail, compounds **2**–**4**, **7**, **9**, and **15** displayed a specific fragment at *m*/*z* 331 ascribable to the aglycon known as patuletin. Compounds **5**, **8**, and **10**–**13** showed a product ion at *m*/*z* 345 related to the aglycon known as spinacetin, while compounds **14** and **17** showed a product ion at *m*/*z* 285 and 359, ascribable to methoxy-apigenin and jaceidin, respectively (Table 1). Compounds **7** and **9**–**13** showed MS/MS spectra ascribable to different phenylpropanoid compounds; analyzing the fragmentation spectra and considering the literature regarding the baby leaves of *S. oleracea*, it was possible to identify compounds **7** and **9**–**13** as glycosylated flavonoids with a feruloyl (*m*/*z* 176) (**9**, **11**, **12**) or coumaroyl (*m*/*z* 147) (**7**, **10**, **13**) moiety (Table 1) [24]. Moreover, through LC-ESI/HRMSMS analysis, compounds **18**–**20** were identified as showing prominent base peaks at *m*/*z* 343, 327, and 357, respectively, attributable to the loss of a glucuronic acid moiety from the parent ion [25]. Therefore, the peaks **18**–**20** were identified as 5,3′,4′-trihydroxy-3-methoxy-6,7-methylenedioxyflavone-4′-*O*-β-glucuronide, 5,4′-dihydroxy-3-methoxy-6,7-methylenedioxiflavone-4′-*O*-β-D-glucuronide, and 5,4′-dihydroxy-3,3′-dimethoxy-6,7-methylenedioxiflavone-4′-*O*-β-glucuronide, respectively (Table 1). Previously, the flavonoid derivatives **2**–**5**, **7**–**17**, and **18**–**20** have been reported in the literature as typical flavonoid compounds of *S. oleracea* leaves. To the best of our knowledge, compound **14** (6-methoxyapigenin-5-methylether) is reported here for the first time in *S. oleracea* [26,27].

The careful analysis of the LC-ESI/HRMSMS spectra of eco-sustainable extracts of *S. oleracea* baby leaves allowed us to characterize numerous compounds belonging to different classes of polar lipids. The detailed analysis of the fragmentation spectra was fundamental for the characterization; in fact, the MS/MS spectra of the individual molecules presented diagnostic product ions of the different classes of polar lipids. The LC-ESI/HRMSMS analysis showed for peaks **21**, **22**, **31**, and **33** pseudomolecular ions at high resolution, ascribable to the molecular formula of C_18_H_32_O_5_, C_18_H_34_O_5_, C_18_H_30_O_3,_ and C_18_H_32_O_3_, respectively, with the fragmentation patterns at *m*/*z* 229 and *m*/*z* 171 attributable to oxylipin derivatives (Table 1). For compound **27**, the LC-ESI/HRMSMS spectrum showed a pseudomolecular ion at *m*/*z* 562.3145 corresponding to the molecular formula C_27_H_50_O_9_NP. The fragmentation pattern of this ion revealed diagnostic fragments of phosphatidylcholines. In detail, compound **27** was characterized by a molecular [(M+FA)-H]^−^ anion formed as an adduct with formic acid and yielding a main [M-15]^−^ product ion, in agreement with the characteristic fragmentation of molecules belonging to this class [28]; furthermore, the presence of the product ion at *m*/*z* 277 allowed the fatty acid to be assigned as C18:3 (Table 1) [28].

Detailed analysis by LC-ESI/HRMSMS allowed us to determine compounds **25**, **28**, **29**, and **32** as sucrose esters with fatty acids. These metabolites showed fragmentation spectra in which the basic peak was represented by the sucrose unit (341 Da) and by a fragment indicative of the nature of the fatty acid. This is the first report of sucrose esters in *S. oleracea* baby leaves (Table 1). Moreover, through LC-ESI/HRMS analysis, compounds **26**, **30**, and **34**–**42** were assigned to the glycolipid class (Table 1). Compounds **30** and **34** were identified as monogalactosyl-monoacylglycerol (MGMG), based on the presence in their MS/MS spectra of the product ion at *m*/*z* 253 corresponding to the monogalactosylglycerol moiety. Furthermore, the LC-ESI/HRMSMS analysis allowed us to assign compound **26** as digalactosyl-monoacylglycerol (DGMG), and compounds **35**–**38**, **40** and **42** as digalactosyl-diacylglycerol (DGDG), considering the presence in their fragmentation spectra of the characteristic product ion at *m*/*z* 397, corresponding to the dehydrated form of the digalactosylglycerol moiety. Finally, compounds **39** and **41**, on the basis of the specific fragmentation pattern, were identified as monogalactosyl diacylglycerol (MGDG) derivatives (Table 1).

### 3.2. PCA of LC-ESI/HRMS Data of S. oleracea Extracts by Targeted Analysis

20-hydroxyecdysone has been suggested to have anabolic properties, promoting protein synthesis and muscle growth; it may enhance strength and muscle mass gains, especially when combined with resistance training. The ability of 20-hydroxyecdysone to promote protein synthesis and muscle repair could help reduce fatigue and expedite recovery after intense physical activity, such as resistance training sessions [14].

Flavonoids are well-known antioxidants that can scavenge free radicals and reduce oxidative stress. By neutralizing reactive oxygen species, flavonoids may protect muscle cells from oxidative damage during exercise, thereby supporting muscle function and recovery; moreover, they have been reported to inhibit lipid peroxidation, which could help to preserve cell membrane integrity and maintain muscle health during exercise [29]. By combining the anabolic effects of 20-hydroxyecdysone with the antioxidant properties of flavonoids, spinach may offer comprehensive support for individuals engaged in resistance training or strenuous physical activity [30].

Herein, a PCA of the LC-ESI/HRMS data obtained for the different eco-sustainable extracts of spinach baby leaves was used to highlight how the extraction method could influence the chemical composition of the extracts [31]. A PCA was performed considering the peak areas corresponding to the different metabolites identified by LC-ESI/HRMS analysis, with the metabolites as the rows of the matrix (variables) and the *S. oleracea* extracts as the columns of the matrix (observations). The resulting model, obtained after scaling the data using the UV (Unit Variance) principle, showed good performance and the absence of anomalous values; in particular, PC1 contributed to 40% of the variance, followed by PC2, which contributed to 22.5%. Therefore, the first two PCs showed a total variance of 62.5%.

**Figure 2:**
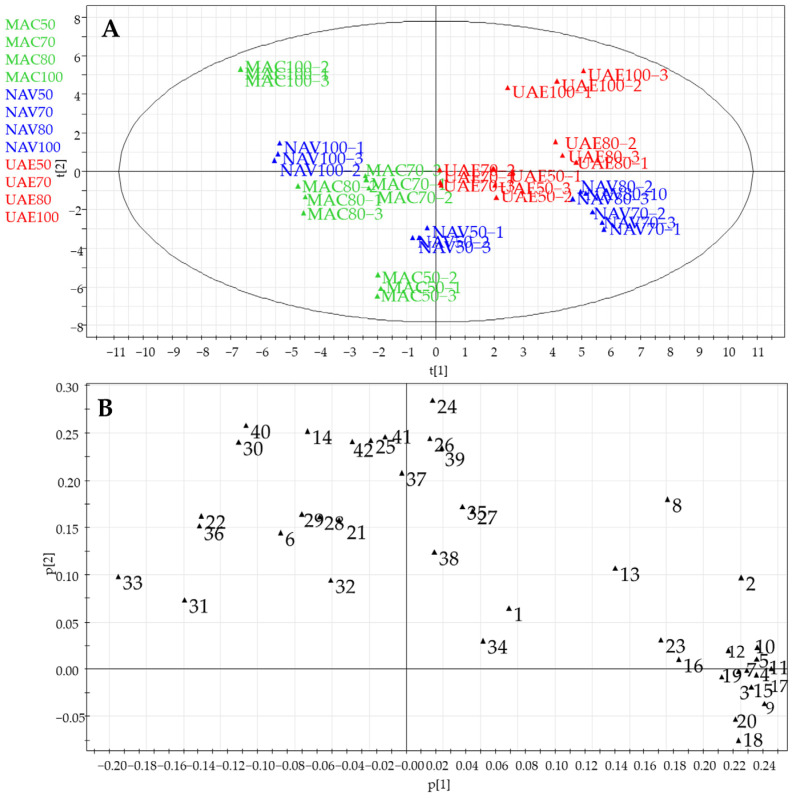
PCA of primary and specialized metabolites in eco-sustainable extracts of spinach baby leaves obtained by LC-ESI/HRMS targeted analysis: (**A**) PCA score scatter plot; (**B**) PCA loading plot. Legend: **1**: allantoin; **2**: Patuletin-3-*O*-β-D-(2″-feruloylglucopyranosyl)-(1→6)-β-D-glucopyranoside; **3**: Patuletin-3-*O*-β-D-glucopyranosyl-(1→6)-[β-D-apiofuranosyl-(1→2)]-β-D-glucopyranoside, **4**: Patuletin-3-*O*-β-D-glucopyranosyl-(1→5)-[β-D-apiofurnaosyl-(1→2)]-β-D-glucopyranoside; **5**: Spinacetin-3-*O*-β-D-glucopyranosyl-(1→6)-[β-D-apiofuranosyl-(1→2)]-β-D-glucopyranoside; **6**: 20-hydroxyecdysone; **7**: Patuletin-3-*O*-β-D-(2″-β-coumaroylglucopyranosyl)-(1→6)-[β-D-apiofuranosyl-(1→2)]-β-D-glucopyranoside; **8**: Spinacetin-3-*O*-β-D-glucopyranosyl-(1→6)-β-D-glucopyranoside; **9**: Patuletin-3-O-β-D-(2″-β-feruloylglucopyranosyl)-(1→6)-[β-D-apiofuranosyl-(1→2)]-β-D-glucopyranoside; **10**: Spinacetin--3-*O*-β-D-(2″-β-coumaroylglucopyranosyl)-(1→6)-[β-D-apiofuranosyl-(1→2)]-β-D-glucopyranoside; **11**: Spinacetin-3-*O*-β-D-(2″-β-feruloylglucopyranosyl)-(1→6)-[β-D-apiofuranosyl-(1→2)]-β-D-glucopyranoside; **12**: Spinacetin-3-*O*-β-D-(2″-feruloylglucopyranosyl)-(1→6)-β-D-glucopyranoside; **13**: Spinacetin-3-*O*-β-D-(2″-coumaroylglucopyranosyl)-(1→6)-β-D-glucopyranoside; **14**: 6-methoxyapigenin-5-methylether; **15**: Patuletin-3-methoxy -4′-*O*-β-D-glucuronide; **16**: Isorhamnetin; **17**: Jaceidin-4′-O-β-D-glucuronide; **18**: 5,3′,4′-trihydroxy-3-methoxy-6:7-methylenedioxyflavone-4′- *O*-β-glucuronide; **19**: 5,4′-dihydroxy-3-methoxy-6:7-methylenedioxiflavone-4′-*O*-β-D-glucuronide; **20**: 5,4′-dihydroxy-3,3′-dimethoxy-6:7-methylenedioxiflavone-4′-*O*-β-glucuronide; **21**: TriHODE; **22**: TriHOME; **23**: Spinasaponia B; **24**: Trimethylellagic acid; **25**: SCMG (18:3); **26**: DGMG (18:3); **27**: l-PC (18:3); **28**: SCMG (18:3); **29**: SCMG (18:2); **30**: MGMG (C18:3); **31**: HOTre; **32**: SCMG (16:2); **33**: HODE; **34**: MGMG (C16:0); **35**: DGDG (C18:3); **36**: DGDG (C18:3, C18:2–10); **37**: DGDG (18:3, C19:3); **38**: DGDG (18:3, 18:3); **39**: MGDG (16:3, 18:2); **40**: DGDG (18:2, 18:3); **41**: MGDG (18:3, 18:3); **42**: DGDG (16:0, 18:3).

The score plot of the principal component analysis displayed a distribution of observations related to the extraction procedure and the kind of solvent used. In detail, the PCA score plot showed a clear difference between the extracts obtained by maceration (MAC) compared to the extracts obtained by ultrasound-assisted extraction (UAE). As observable in Figure 2A, the UAE extracts are distributed in the right quadrants and the MAC extracts in the left quadrants of the score plot, separated by the first principal component.

Moreover, Figure 2A highlights that the EtOH extracts obtained by using SLDE and maceration were separated by the second component from the extracts of EtOH:H_2_O obtained using the same extractions method. Consequently, the PCA score plot highlights that both the solvent and the extraction method could influence the kind of metabolites extracted. To obtain information relating to the type of metabolites responsible for the distribution on the score plot, careful analysis of the PCA loading plot graph was necessary. In particular, EtOH extracts obtained by using maceration (MAC) and SLDE-Naviglio (NAV) were mainly characterized by the presence of 20-hydroxyecdysone (**6**), recognized as the “natural anabolic agent” of spinach. Moreover, the EtOH extracts were richest in polar lipid derivatives (**21**, **22**, **24**–**42**), which contribute to the overall chemical composition of the extracts and may have diverse biological activities. Hydroalcoholic extracts, particularly those obtained by using SLDE-Naviglio with EtOH:H_2_O (80:20 and 70:30, *v*/*v*), were found to be the richest in flavonoid derivatives (**3**–**5**, **7**–**11**, **14**, **15**–**17**, **18**–**20**). Flavonoids are well-known phytochemicals with antioxidant properties and are considered major contributors to the antioxidant activity of spinach [26,32]. These flavonoid derivatives play a crucial role in scavenging free radicals and protecting cells from oxidative damage, thereby potentially conferring various health benefits, including reducing the risk of chronic diseases and promoting overall well-being.

### 3.3. Isolation of Main Specialized Metabolites of Extracts of S. oleracea Baby Leaves

**Figure 3:**
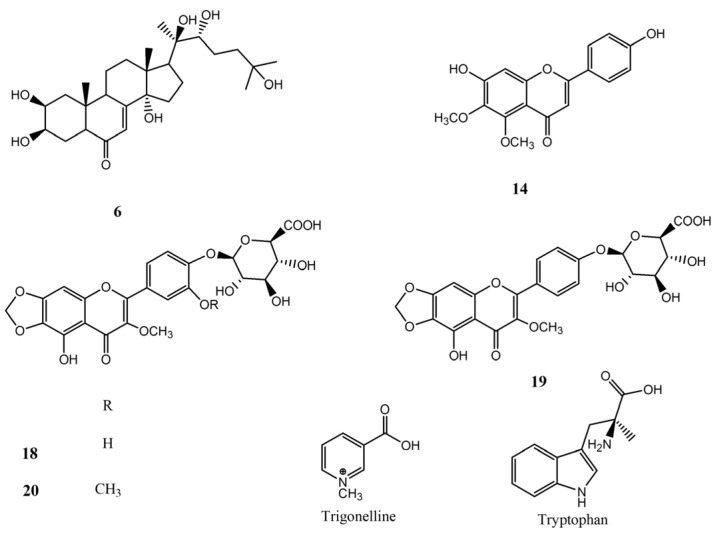
Bioactives isolated from *S. oleracea* baby leaves. Legend: **6**: 20-hydroxyecdysone; **14**: 6-methoxyapigenin-5-methylether; **18**: 5,3′,4′-trihydroxy-3-methoxy-6:7-methylenedioxyflavone-4′-*O*-β-glucuronide; **19**: 5,4′-dihydroxy-3-methoxy-6:7-methylenedioxiflavone-4′-*O*-β-D-glucuronide; **20**: 5,4′-dihydroxy-3,3′-dimethoxy-6:7-methylenedioxiflavone-4′-*O*-β-glucuronide.

In order to unambiguously identify the main specialized metabolites occurring in eco-sustainable extracts of *S. oleracea* baby leaves, a phytochemical analysis has been carried out. PCA of spinach baby leaf extracts obtained by LC-ESI/HRMS targeted analysis suggested that the extract more interesting in terms of flavonoid derivatives was EtOH:H_2_O (70:30, *v*/*v*), obtained by using SLDE-Naviglio; therefore; the above-mentioned extract was selected for the phytochemical investigation. The careful study of mono- and bidimensional NMR experiments of purified compounds allowed us to clearly identify specialized metabolites such as **6**, **14**, and **18**–**20**. As evidenced by the LC-ESI/HRMS profile, 20-hydroxyecdysone (**6**) and methylenedioxyflavone derivatives (**18**–**20**) represent the most abundant compounds of *S. oleracea* baby leaves [33] (Figure 3).

### 3.4. Fingerprint of Eco-Sustainable Extracts of S. oleracea by ^1^H-NMR Analysis

**Figure 4:**
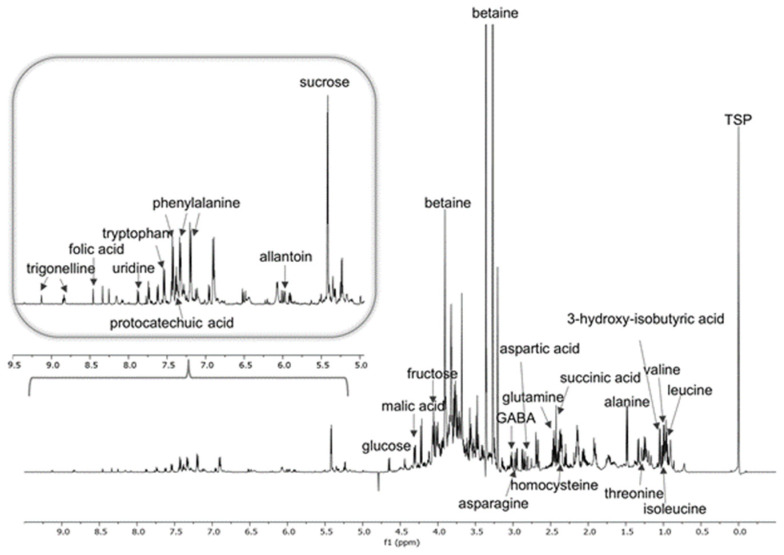
^1^H NMR spectrum with annotations of identified metabolites detected in *S. oleracea* EtOH:H_2_O 70:30 *v*/*v* extract obtained by using SLDE-Naviglio.

In order to obtain complete information about the primary metabolites, such as amino acids and carbohydrates, which contribute to the nutritional and bioactivity profile of the eco-sustainable extracts of *S. oleracea* baby leaves, a multivariate analysis approach based on ^1^H-NMR was performed. In fact, the concentration of metabolites was calculated by comparing the intensity of each “key” peak of each metabolite with the peak relating to the internal standard; the software Chenomx database (Chenomx NMR Suite version 10.0) allowed us to identify 24 compounds, including amino acids, carbohydrates, and organic acids. In particular, aliphatic and derived amino acids, such as γ-amino butyric acid (GABA), aspartic acid, asparagine, alanine, isoleucine, leucine, valine, glutamine, homocysteine, threonine, and betaine were observed in the amino acid region between 0.91 and 3.90 ppm. Aromatic amino acids such as phenylalanine, tryptophan, and tyramine were assigned to 7.32, 7.54, and 7.25 ppm, respectively (Table S1). Carbohydrates such as fructose, sucrose, and glucose were attributed by observing specific chemical shifts in the sugar area, between 4.01 and 5.38 ppm (Figure 4 and Figure S1, Table S1). Furthermore, on the basis of the analysis of the ^1^H-NMR spectra, it was possible to assign specific signals of the nitrogenous base uridine (7.91 ppm) and various organic acids such as folic acid (7.55 ppm), 3-hydroxybutyric acid (1.12 ppm), malic acid (4.29 ppm), protocatechuic acid (7.41 ppm), and succinic acid (2.43 ppm). Furthermore, the chemical shifts at 6.01 and 9.11 ppm were attributed to allantoin and trigonelline, respectively (Figure 4, Table S1).

The relative abundance of each metabolite (expressed as mM) has been determined by using Chenomx 10.0 software [34,35]. Herein, the q-NMR results highlighted the non-essential amino acid betaine as the most abundant compound in the extracts in the range of −1.88–28.42 mM with the highest concentration in the EtOH:H_2_O (70:30, *v*/*v*) extract obtained by using SLDE-Naviglio (Table 2). This result is particularly interesting since antioxidant, neuroprotective, and cardioprotective properties have been reported for betaine, along with an ability to increase strength and sports performance [36]. Among essential amino acids, asparagine exhibited the highest concentration in the extracts EtOH:H_2_O 70:30 *v*/*v* and 50:50 *v*/*v* obtained by using SLDE-Naviglio (5.00 and 4.46 mM, respectively) (Table 2). Finally, carbohydrates, according to the polarity of these compounds, were mainly present in the EtOH:H_2_O extracts. In particular, fructose and sucrose displayed the highest concentration in the EtOH:H_2_O (50:50, *v*/*v*) extracts obtained by using maceration (1.91 mM) and EtOH:H_2_O (70:30, *v*/*v*) obtained by using UAE (2.35 mM), respectively (Table 2).

### 3.5. PCA of ^1^H-NMR Data Obtained for Different Extracts

**Figure 5:**
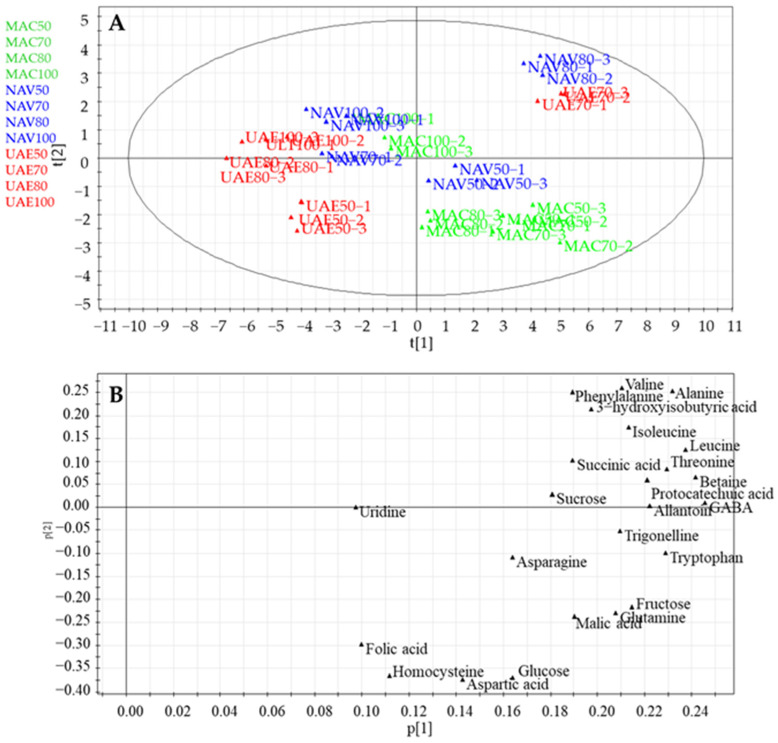
PCA primary metabolites in spinach eco-sustainable extracts obtained by ^1^H-NMR targeted analysis: (**A**) PCA score scatter plot; (**B**) PCA loading plot.

Successively, ^1^H NMR-PCA was performed to explore the influence of the different solvents and methods on the extraction of the primary metabolites (Figure 5). The concentrations of each metabolite identified in the ^1^H NMR spectra were considered as variables (rows), while observations were represented by the different extracts of *S. orelacea* (columns). The Pareto scaling principle was used and showed good results and the absence of outliers. PC1 contributed to 59.0% of the variance, followed by PC2, which contributed 14.0% (total variance of 73.0%). The PCA score plot diagram (Figure 5A) shows a difference between the extracts due to the solvent used rather than the extraction method. In particular, the PCA score plot showed that all EtOH extracts were located close together in the upper left quadrant, while the extracts obtained by using hydroalcoholic mixtures were mainly located in the right of the PCA score plot. The PCA loading plot (Figure 5B) shows a higher abundance of primary metabolites in the hydroalcoholic extracts obtained by using SLDE-Naviglio and maceration. In particular, the extracts mentioned above were richer than the ethanol extracts and the UAE extracts in terms of the content of amino acids, carbohydrates, and organic acid derivatives.

### 3.6. Evaluation of Total Phenolic Content, Flavonoid Content, and Radical Scavenging Activity of Eco-Sustainable Extracts of S. oleracea Baby Leaves

Eco-sustainable extracts of *S. oleracea* baby leaves showed a phenolic content in the range of 118.35–206.60 mg GAE/g (milligrams of gallic acid equivalents per gram of dry extract). The high phenolic content displayed by the EtOH:H_2_O (80:20, *v*/*v*) and EtOH:H_2_O (70:30, *v*/*v*) extracts obtained by using SLDE-Naviglio, with a value of 181.84 and 206.60 mg GAE/g, respectively (Table 3), could be ascribed to the synergic effect of the solvent mixture. Concerning the estimation of the flavonoid content, the same extracts exhibited the highest flavonoid content (Table 3). Therefore, the extract EtOH:H_2_O (70:30, *v*/*v*) obtained by using SLDE-Naviglio showed the highest radical scavenging activity with a TEAC value of 2.05 mM, according to the phenolic and flavonoid content. Total flavonoid and TEAC values of the extracts against their corresponding total phenolic content (TPC) were evaluated using Pearson’s method. As previously reported, Pearson’s correlation is significantly positive if 0.61 ≤ r^2^ ≤ 0.97. In detail, there was a positive correlation between the total flavonoid value and TPC (r^2^ = 0.88), between TEAC and TPC (r^2^ = 0.87), and between the flavonoid value and TEAC (r^2^ = 0.80) (Table 3)

### 3.7. Quantitative Analysis of 20-Hydroxyecdysone *(**6**)* in Extracts of S. oleracea

In the last few years, 20-hydroxyecdysone has gained attention as a dietary supplement provided to athletes for its supposed ability to enhance force and muscle mass during sports, reduce fatigue, and ease recovery [13,37].

**Figure 6:**
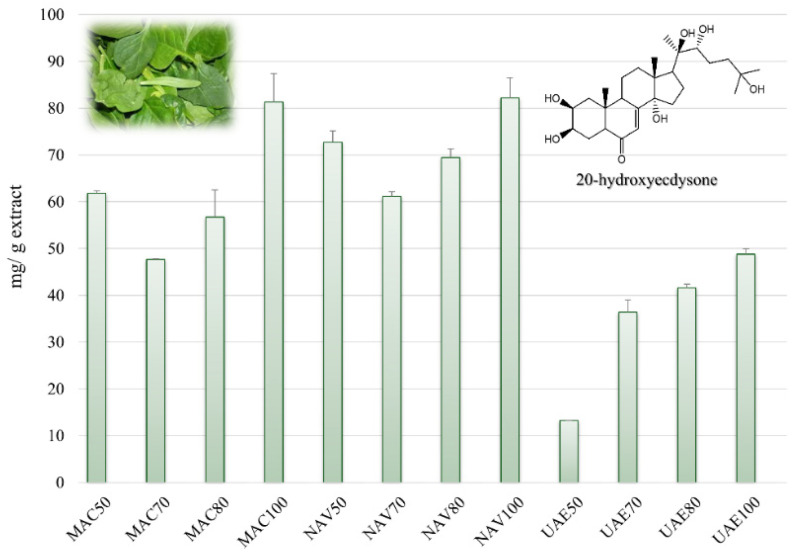
Quantitative determination of 20-hydroxyecdysone (mg/g extract ± SD) in extracts of *S. oleracea* baby leaves.

Therefore, 20-hydroxyecdysone (**6**) was quantified in all of the extracts of *S. oleracea* baby leaves by LC-ESI/QTrap/MS/MS analysis using the MRM method, where specific precursor ions are selected in the first quadrupole and fragmented in the collision cell, giving rise to specific product ions which are monitored in the third quadrupole. In our case, 20-hydroxyecdysone (**6**) showed a pseudomolecular ion [(M+FA)-H]^−^ at *m*/*z* 525, which generated a specific fragment at *m*/*z* 319, resulting from the cleavage of the side chain among C17-C20; consequently, this diagnostic transition was selected for MRM analysis. Based on the selected transition, the amount (mg/g of extract) of 20-hydroxyecdysone (**6**) in the extracts was determined. It occurred in a concentration range of 13.26–82.16 mg/g extract, showing the highest concentration in the EtOH extracts obtained by using SLDE-Naviglio (82.16 mg/g extract) and maceration (81.27 mg/g extract) (Figure 6) according to the PCA loading plot results (Figure 2).

## 4. Conclusions

The optimization of conditions for the extraction of bioactives from *Spinacia oleracea* L. cultivar platypus baby leaves is crucial for maximizing the yield of target metabolites such as 20-hydroxyecdysone and flavonoid derivatives. Herein, for the first time for this species, various eco-sustainable extraction solvents, including EtOH and EtOH:H_2_O mixtures, and techniques including maceration at room temperature, solid–liquid dynamic extraction (SLDE-Naviglio), and UAE were used. The LC-ESI/HRMS analysis identified 42 metabolites in *S. oleracea* L. cultivar platypus baby leaves, including flavonoids, terpenoids, oxylipins, and glycolipids. Among these metabolites, 20-hydroxyecdysone and the main flavonoids were further purified and characterized using 1D and 2D-NMR, in combination with FIA-MS.

The metabolomic approach based on the LC-ESI/HRMS results revealed distinct chemical compositions of EtOH and hydroalcoholic extracts from *S. oleracea* baby leaves, with EtOH extracts, mainly extracts obtained by using SLDE-Naviglio, being characterized by 20-hydroxyecdysone and polar lipid derivatives, while hydroalcoholic extracts, mainly the EtOH:H_2_O (70:30, *v*/*v*) extract obtained by using SLDE-Naviglio, being enriched with flavonoid derivatives. These findings provide valuable insights into the chemical diversity and potential health-promoting properties of spinach extracts, highlighting the importance of extraction methods and solvents in targeting specific bioactive compounds. In addition, the ^1^H NMR analysis of eco-sustainable extracts of *S. oleracea* baby leaves identified a total of 24 primary metabolites, including amino acids, carbohydrates, and organic acid derivatives. Among these metabolites, the EtOH:H_2_O (70:30, *v*/*v*) extract obtained by using solid–liquid dynamic extraction (SLDE-Naviglio) was found to have the highest concentration of betaine, measured at 28.42 mM. This result is particularly interesting considering that this extract was the richest in flavonoids and that betaine is a primary metabolite known for its antioxidant, neuroprotective, and cardioprotective properties. It has also been reported to increase strength and sports performance. Considering the commercial value of 20-hydroxyecdysone, LC-ESI/QTrap/MS/MS with the MRM method was employed to quantify 20-hydroxyecdysone in all eco-sustainable extracts. The results revealed that the highest amount was in the EtOH extract obtained by using SLDE-Naviglio (82.16 mg/g extract). This finding underscores the effectiveness of SLDE-Naviglio in extracting 20-hydroxyecdysone from *S. oleracea* baby leaves, and it suggests the potential utility of this extract in health and wellness due to the high content of this bioactive compound.

## Figures and Tables

**Table 1:** Retention times (R*_t_*), Δ ppm, molecular formula, [M−H]^−^, and MS/MS values of compounds occurring in baby leaves of *S. oleracea* EtOH (100%) extract (SLDE-Naviglio) determined by using high-resolution LC-ESI/HRMSMS (negative ion mode).

	R*_t_* (min)	Compound	Molecular Formula	[M−H]^−^	Δppm	MS/MS
**1**	1.71	allantoin	C_4_H_6_N_4_O_3_	157.0358	0.92	114.0296 (C_4_H_6_N_2_O_2_),
**2**	10.89	patuletin-3-*O*-β-D-(2″-feruloylglucopyranosyl)-(1→6)-β-D-glucopyranoside	C_31_H_44_O_26_	831.1992	−5.47	655.1519 (C_28_H_31_O_18_), 331.0451 (C_16_H_11_O_8_)
**3**	12.79	patuletin-3-*O*-β-D-glucopyranosyl-(1→6)-[β-D-apiofuranosyl-(1→2)]-β-D-glucopyranoside	C_33_H_40_O_22_	787.1937	1.25	331.0457 (C_16_H_11_O_8_)
**4**	13.27	patuletin-3-*O*-β-D-glucopyranosyl-(1→5)-[β-D-apiofuranosyl-(1→2)]-β-D-glucopyranoside	C_51_H_32_O_8_	771.1996	−1.69	331.0449 (C_16_H_11_O_8_)
**5**	13.31	spinacetin-3-*O*-β-D-glucopyranosyl-(1→6)-[β-D-apiofuranosyl-(1→2)]-β-D-glucopyranoside	C_52_H_34_O_9_	801.2090	0.81	345.0608 (C_17_H_13_O_8_), 329.0303 (C_16_H_9_O_8_)
**6**	13.39	20-hydroxyecdysone	C_28_H_46_O_9_	525.3069 *	3.33	479.3014 (C_27_H_43_O_7_), 319.1911 (C_19_H_27_O_4_), 159.1016 (C_8_H_15_O_3_)
**7**	13.95	patuletin-3-*O*-β-D-(2″-β-coumaroylglucopyranosyl)-(1→6)-[β-D-apiofuranosyl-(1→2)]-β-D-glucopyranoside	C_60_H_38_O_11_	933.2311	−2.10	331.0440 (C_16_H_11_O_8_), 315.0149 (C_15_H_7_O_8_)
**8**	14.00	spinacetin-3-*O*-β-D-glucopyranosyl-(1→6)-β-D-glucopyranoside	C_29_H_34_O_18_	669.1673	1.73	345.0616 (C_17_H_13_O_8_), 331.0417 (C_16_H_11_O_8_)
**9**	14.04	patuletin-3-*O*-β-D-(2″-β-feruloylglucopyranosyl)-(1→6)-[β-D-apiofuranosyl-(1→2)]-β-D-glucopyranoside	C_43_H_48_O_25_	963.2413	1.22	331.0444 (C_16_H_11_O_8_), 315.0147 (C_15_H_7_O_8_)
**10**	14.43	spinacetin--3-*O*-β-D-(2″-β-coumaroylglucopyranosyl)-(1→6)-[β-D-apiofuranosyl-(1→2)]-β-D-glucopyranoside	C_36_H_52_O_29_	947.2465	1.35	345.0619 (C_17_H_13_O_8_), 331.0407 (C_16_H_11_O_8_)
**11**	14.47	spinacetin-3-*O*-β-D-(2″-β-feruloylglucopyranosyl)-(1→6)-[β-D-apiofurnaosyl-(1→2)]-β-D-glucopyranoside	C_37_H_54_O_30_	977.2575	1.81	345.0605 (C_17_H_13_O_8_), 330.0378 (C_16_H_10_O_8_)
**12**	15.33	spinacetin-3-*O*-β-D-(2″-feruloylglucopyranosyl)-(1→6)-β-D-glucopyranoside	C_32_H_46_O_26_	845.2165	−3.32	345.0614 (C_17_H_13_O_8_), 330.0381 (C_16_H_10_O_8_)
**13**	15.50	spinacetin-3-*O*-β-D-(2″-coumaroylglucopyranosyl)-(1→6)-β-D-glucopyranoside	C_31_H_44_O_25_	815.2040	−5.03	345.0613 (C_17_H_13_O_8_), 330.0781 (C_16_H_10_O_8_)
**14**	15.81	6 metoxyapigenin-5-methylether	C_18_H_16_O_8_	359.0775 *	3.80	285.2070 (C_15_H_9_O_6_)
**15**	15.85	satuletin-3-methoxy-4′-*O*-β-D-glucuronide	C_23_H_22_O_14_	521.0938	2.36	345.0617 (C_17_H_13_O_8_), 331.0381 (C_16_H_11_O_8_), 315.0149 (C_15_H_7_O_8_)
**16**	16.24	isorhamnetin	C_16_H_12_O_7_	315.0590	2.38	301.1105 (C_15_H_9_O_7_)
**17**	16.24	jaceidin-4′-*O*-β-D-glucuronide	C_24_H_24_O_14_	535.1093	2.01	359.0775 (C_18_H_15_O_8_), 329.0302 (C_16_H_9_O_8_)
**18**	16.80	5,3′,4′-trihydroxy-3-methoxy-6:7-methylenedioxyflavone-4′-*O*-β-glucuronide	C_23_H_20_O_14_	519.0779	1.83	343.0460 (C_17_H_11_O_8_), 328.0225 (C_16_H_8_O_8_)
**19**	17.45	5,4′-dihydroxy-3-methoxy-6:7-methylenedioxiflavone-4′-*O*-β-D-glucuronide	C_23_H_20_O_13_	503.0830	1.42	327.0509 (C_17_H_11_O_7_), 312.0276 (C_16_H_8_O_7_)
**20**	17.73	5,4′-dihydroxy-3,3′-dimethoxy-6:7-methylenedioxiflavone-4′-*O*-β-glucuronide	C_24_H_22_O_14_	533.0933	1.38	357.0617 (C_18_H_13_O_8_), 327.0145 (C_16_H_7_O_8_)
**21**	18.05	TriHODE	C_18_H_32_O_5_	327.2178	3.76	229.1441 (C_12_H_21_O_4_), 171.1018 (C_9_H_15_O_3_)
**22**	18.83	TriHOME	C_18_H_34_O_5_	329.2338	4.49	229.1144 (C_12_H_21_O_4_), 171.1018 (C_9_H_15_O_3_)
**23**	19.34	spinasaponin B	C_42_H_66_O_15_	809.4330	1.24	473.2382 (C_23_H_37_O_10_)
**24**	20.17	trimethylellagic acid	C_17_H_12_O_8_	343.0461	3.60	328.0224 (C_16_H_8_O_8_), 299.0195 (C_15_H_7_O_7_)
**25**	20.98	SCMG (18:3)	C_31_H_52_O_14_	647.3291 *	1.77	341.1089 (C_12_H_21_O_11_), 277.2172 (C_18_H_29_O_2_)
**26**	22.71	DGMG (18:3)	C_34_H_58_O_16_	721.3647 *	0.64	397.1353 (C_15_H_25_O_12_), 277.2173 (C_18_H_29_O_2_)
**27**	23.23	l-PC (18:3)	C_27_H_50_O_9_NP	562.3145 *	0.56	502.2941 (C_29_H_42_O_7_), 277.2171 (C_18_H_29_O_2_)
**28**	23.57	SCMG (18:3)	C_31_H_52_O_14_	647.3289 *	1.53	341.1089 (C_12_H_21_O_11_), 277.2172 (C_18_H_29_O_2_)
**29**	23.87	SCMG (18:2)	C_31_H_54_O_14_	649.3442 *	1.84	341.1093 (C_12_H_21_O_11_), 279.2328 (C_18_H_31_O_2_)
**30**	24.65	MGMG (C18:3)	C_28_H_48_O_11_	559.3125 *	3.92	277.2173 (C_18_H_29_O_2_), 253.0927 (C_9_H_17_O_8_)
**31**	25.08	HOTre	C_18_H_30_O_3_	293.2120	0.92	171.1018 (C_9_H_15_O_3_)
**32**	25.94	SCMG (16:2)	C_28_H_52_O_12_	579.3387	1.25	341.1079 (C_12_H_21_O_11_), 255.2328 (C_16_H_31_O_2_)
**33**	26.60	HODE	C_18_H_32_O_3_	295.2284	0.71	295.2277 (C_18_H_31_O_3_), 277.2171 (C_18_H_29_O_2_), 171.1017 (C_9_H_15_O_3_)
**34**	27.02	MGMG (C16:0)	C_25_H_48_O_9_	491.3222	1.51	255.2329 (C_16_H_31_O_2_)
**35**	27.62	DGDG (C18:3)	C_25_H_45_O_8_P	503.2759	1.77	277.2173 (C_18_H_29_O_2_)
**36**	30.77	DGDG (C18:3, C18:2–10)	C_51_H_84_O_16_	997.5746 *	1.73	397.1353 (C_15_H_25_O_12_), 277.2172 (C_18_H_29_O_2_)
**37**	34.27	DGDG (18:3, C19:3)	C_49_H_80_O_15_	907.5429	0.53	397.1088 (C_15_H_25_O_12_), 341.1088 (C_12_H_21_O_11_), 291.2331 (C_19_H_31_O_2_), 277.2171 (C_18_H_29_O_2_)
**38**	36.81	DGDG (18:3, 18:3)	C_51_H_84_O_15_	935.5743	1.81	397.1353 (C_15_H_25_O_12_), 277.2172 (C_18_H_29_O_2_)
**39**	37.68	MGDG (16:3, 18:2)	C_43_H_70_O_10_	745.4893	0.99	490.3276 (C_29_H_46_O_6_), 277.2172 (C_18_H_29_O_2_), 249.1859 (C_16_H_25_O_2_)
**40**	38.06	DGDG (18:2, 18:3)	C_51_H_86_O_15_	937.5895	1.26	397.1341 (C_15_H_25_O_12_), 279.2332 (C_18_H_31_O_2_), 277.2172 (C_18_H_29_O_2_)
**41**	40.31	MGDG (18:3, 18:3)	C_45_H_74_O_10_	773.5209	1.35	277.2173 (C_18_H_29_O_2_)
**42**	41.17	DGDG (16:0, 18:3)	C_49_H_86_O_15_	913.5892	1.02	397.1352 (C_15_H_25_O_12_), 277.2173 (C_18_H_29_O_2_), 255.2329 (C_16_H_31_O_2_)

* *m*/*z* refered to [(M+FA)−H]^−^. TriHODE: Trihydroxy-OctadecaDiEnoic acid; TriHOME: Trihydroxy-OctadecEnoic acid; SCMG: sucrose-monoacylglycerol; DGMG: digalactosyl-monoacylglycerol; l-PC: lyso-phosphatidyl-choline; MGMG: monogalactosyl-monoacylglycerol; HoTre: Hydroxy-OctadecaTriEnoic acid; HODE: Hydroxy-OctadecaDiEnoic acid; DGDG: digalactosyl-diacylglycerol.

**Table 2:** Relative abundance concentration (expressed in mM ± SD) of amino acids, sugars, organic acids, and other primary metabolites of eco-sustainable extracts of *S. oleracea* baby leaves by ^1^H-NMR analysis.

Extract	MAC50 ± SD		MAC70 ± SD		MAC80 ± SD		MAC100 ± SD		NAV50 ± SD		NAV70 ± SD		NAV80 ± SD		NAV100 ± SD		UAE50 ± SD		UAE70 ± SD		UAE80 ± SD		UAE100 ± SD	
**amino acid derivatives**																								
**alanine**	0.32 *	0.02	0.26	0.04	0.20 *	0.02	0.23 *	0.02	0.27 *	0.02	1.12 *	0.14	0.60 *	0.03	0.17 *	0.01	0.10 *	0.02	0.65 *	0.08	0.23 *	0.01	0.08 *	0.01
**asparagine**	0.80 *	0.06	0.96	0.22	0.93 *	0.07	0.36 *	0.09	4.46 **	0.03	5.00 *	0.96	3.27 *	0.16	0.26 *	0.10	0.84 *	0.01	1.59 *	0.04	0.51 *	0.04	0.26 *	0.04
**aspartic acid**	0.93 *	0.03	0.94	0.10	0.85 *	0.05	0.18 *	0.08	0.37 *	0.03	2.38 *	0.77	1.07 *	0.11	0.10 *	0.03	0.71 *	0.01	1.17 *	0.13	0.44 *	0.02	0.28 *	0.10
**betaine**	16.23 *	3.94	18.21 **	0.21	13.60 **	0.16	12.95 *	0.99	15.27 **	0.86	28.42 *	4.04	15.52 *	2.08	16.28 **	0.57	1.88 *	0.05	8.07 *	1.45	7.28 *	0.07	6.51 *	0.05
**glutamine**	0.66 *	0.05	0.59 *	0.07	0.55 *	0.03	0.27 *	0.04	0.31 *	0.03	2.65 *	0.61	1.28 *	0.09	0.12 *	0.03	0.17 *	0.03	0.72 *	0.07	0.17 *	0.01	0.10 *	0.03
**isoleucine**	0.40 *	0.03	0.28 *	0.07	0.16 *	0.01	0.22 *	0.03	0.23 *	0.02	0.97 *	0.06	0.30 *	0.04	0.26 *	0.01	0.05 *	0.01	0.62 *	0.10	0.21 *	0.01	0.11 *	0.06
**leucine**	0.46 *	0.02	0.36 *	0.03	0.24 *	0.04	0.24 *	0.02	0.33 *	0.04	0.78 *	0.07	0.42 *	0.01	0.21 *	0.01	0.08 *	0.01	0.79 *	0.09	0.28 *	0.03	0.11 *	0.01
**phenylalanine**	0.17 *	0.01	0.17 *	0.02	0.10 *	0.01	0.11 *	0.01	0.14 *	0.01	1.27 *	0.51	0.44 *	0.14	0.09 *	0.01	0.05 *	0.01	0.34 *	0.01	0.09 *	0.01	0.10 *	0.01
**threonine**	0.31 *	0.03	0.25 *	0.03	0.19 *	0.01	0.18 *	0.04	0.37 *	0.06	0.56 *	0.06	0.31 *	0.02	0.15 *	0.01	0.09 *	0.01	0.66 *	0.05	0.24 *	0.01	0.08 *	0.02
**tryptophan**	0.18 *	0.02	0.21 *	0.03	0.15 *	0.02	0.08 *	0.02	0.15 *	0.02	1.37 *	0.13	0.53 *	0.09	0.08 *	0.02	0.06 *	0.01	0.25 *	0.06	0.07 *	0.01	0.07 *	0.04
**valine**	0.17 *	0.02	0.20 *	0.07	0.17 *	0.01	0.21 *	0.01	0.20 *	0.01	0.46 *	0.10	0.29 *	0.03	0.17 *	0.01	0.04 *	0.01	0.46 *	0.04	0.15 *	0.02	0.07	0.01
**sugars**																								
**fructose**	1.91 *	0.55	1.55 *	0.32	1.32 *	0.25	0.66 *	0.04	1.49 *	0.14	1.83 *	0.33	1.64 *	0.47	0.44 *	0.04	0.98 *	0.07	1.37 *	0.39	0.68 *	0.09	0.36 *	0.06
**glucose**	0.40 *	0.04	0.37 *	0.05	0.26 *	0.01	0.16 *	0.03	0.33 *	0.03	0.65 *	0.01	0.36 *	0.09	0.08 *	0.02	0.13 *	0.02	0.36 *	0.05	0.04 *	0.01	0.07 *	0.01
**sucrose**	0.66 *	0.04	1.01 *	0.05	0.80 *	0.02	0.78 *	0.06	0.83 *	0.06	1.86 *	0.08	1.78 **	0.19	0.44 *	0.04	0.97 *	0.01	2.35 *	0.36	0.87 *	0.02	0.47 *	0.01
**organic acids**																								
**malic acid**	1.40 **	0.07	1.37 *	0.08	1.13 *	0.03	0.28 *	0.04	0.76 *	0.04	3.27 *	0.81	1.89 *	0.15	0.17 *	0.05	1.22 *	0.07	2.09 *	0.34	0.55 *	0.01	0.31 *	0.03
**protocatechuic acid**	0.17 *	0.01	0.25 *	0.04	0.16 *	0.03	0.14 *	0.02	0.14 *	0.02	0.67 *	0.01	0.44 *	0.05	0.11 *	0.03	0.09 *	0.02	0.35 *	0.05	0.11 *	0.02	0.07 *	0.02
**succinic acid**	0.16 *	0.01	0.07 *	0.02	0.15 *	0.01	0.15 *	0.01	0.13 *	0.02	0.19 *	0.01	0.13 *	0.01	0.06 *	0.01	0.06 *	0.01	0.29 *	0.03	0.09 *	0.01	0.05 *	0.01
**other primary metabolites**																								
**allantoin**	0.63 *	0.03	0.74 *	0.03	0.38 *	0.05	0.55 *	0.10	0.74 *	0.04	2.71 *	0.62	1.29 *	0.78	0.40 *	0.04	0.17 *	0.01	0.95 *	0.02	0.27 *	0.02	0.17 *	0.04
**folic acid**	0.10 *	0.06	0.33 *	0.05	0.10 *	0.02	0.07 *	0.04	0.08 *	0.02	1.33 *	0.91	0.34 *	0.07	0.04 *	0.02	0.03 *	0.02	0.09 *	0.02	0.12 *	0.02	0.12 *	0.01
**GABA**	0.33 *	0.04	0.32 *	0.06	0.21 *	0.02	0.22 *	0.02	0.23 *	0.02	1.70 *	0.77	0.64 *	0.17	0.18 *	0.02	0.12 *	0.02	0.62 *	0.08	0.12 *	0.01	0.09 *	0.01
**3-hydroxyisobutyric acid**	0.22 *	0.01	0.25 *	0.03	0.18 *	0.01	0.16 *	0.04	0.11 *	0.02	0.38 *	0.03	0.35 *	0.02	0.13 *	0.01	0.05 *	0.01	0.39 *	0.07	0.17 *	0.05	0.04 *	0.01
**homocysteine**	1.01 *	0.11	0.75 *	0.03	0.91 *	0.22	0.58 *	0.13	0.97 *	0.09	0.87 *	0.07	0.59 *	0.08	0.15 *	0.06	0.22 *	0.01	0.24 *	0.05	0.22 *	0.01	0.06 *	0.02
**trigonelline**	0.08 *	0.02	0.13 *	0.07	0.06 *	0.03	0.07 *	0.01	0.07 *	0.02	0.53 *	0.05	0.21 *	0.02	0.05 *	0.02	0.02 *	0.01	0.13 *	0.03	0.05 *	0.02	0.03 *	0.01
**tyramine**	0.14 *	0.01	0.17 *	0.03	0.09 *	0.01	0.08 *	0.02	0.11 *	0.01	0.73 *	0.07	0.41 *	0.09	0.04 *	0.01	0.05 *	0.01	0.30 *	0.02	0.08 *	0.01	0.04 *	0.01
**uridine**	0.06 *	0.02	0.08 *	0.01	0.05 *	0.01	0.04 *	0.01	0.17 *	0.01	0.05 *	0.01	0.18 *	0.01	0.09 *	0.01	0.05 *	0.01	0.09 *	0.02	0.02 *	0.01	0.02 *	0.01

MAC: maceration; NAV: SLDE-Naviglio; UAE: ultrasound-assisted extraction; * *p* < 0.05; ** *p* < 0.001.

**Table 3:** Phenolic content, total flavonoid content, and TEAC of eco-sustainable extracts of baby leaves of *S. oleracea* L.

Extract	Total Phenol Content ^a^ (mg GAE/g ± SD ^b^)	Total Flavonoid Content ^c^ (mg rutin/g ± SD ^b^)	TEAC ^d^ (mg/mL ± SD ^b^)
**MAC50**	123.43 ± 0.95 ***	12.72 ± 0.66 ***	1.79 ± 0.12 ***
**MAC70**	130.73 ± 0.55 ***	10.90 ± 0.13 ***	1.76 ± 0.13 ***
**MAC80**	142.48 ± 0.95 ***	25.45 ± 0.13 ***	1.74 ± 0.10 ***
**MAC100**	119.94 ± 1.10 ***	11.20 ± 0.10 **	1.63 ± 0.09 ***
**NAV50**	139.62 ± 0.55 ***	27.87 ± 0.14 ***	1.77 ± 0.13 ***
**NAV70**	206.60 ± 5.50 ***	41.05 ± 0.15 ***	2.05 ± 0.23 ***
**NAV80**	181.84 ± 0.10 ***	40.14 ± 0.13 ***	1.91 ± 0.10 ***
**NAV100**	118.35 ± 0.55 **	14.08 ± 0.23 ***	1.73 ± 0.05 ***
**UAE50**	157.40 ± 0.55 ***	24.31 ± 0.13 ***	1.77 ± 0.12 ***
**UAE70**	166.28 ± 1.46 ***	23.02 ± 0.23 ***	1.75 ± 0.09 ***
**UAE80**	139.62 ± 3.30 ***	15.96 ± 0.35 ***	1.74 ± 0.08 ***
**UAE100**	120.41 ± 3.85 ***	11.98 ± 0.45 **	1.76 ± 0.09 ***

^a^ Values are expressed as milligrams of gallic acid equivalents (GAE) per gram of dried extract (mg GAE/g dried extract); ^b^ SD: the results are expressed as the mean of three experiments; SD, standard deviation; ^c^ values are expressed as milligrams of rutin equivalents per gram of dried extract (mg rutin/g dried extract); ^d^ values are expressed as the concentration (mM) of a standard Trolox solution exerting the same antioxidant activity of a 1 mg/mL solution of the tested extract, with the concentration of extracts being 0.25–1.0 mg/mL. ** *p* < 0.002 and *** *p* < 0.001 vs. control according to a one-way ANOVA followed by Dunnett’s multiple comparison test.

## Data Availability

The original contributions presented in the study are included in the article/Supplementary Materials, further inquiries can be directed to the corresponding author.

## Supplementary Materials

The following supporting information can be downloaded at: https://www.mdpi.com/article/10.3390/foods13223699/s1, Table S1: Characteristic ^1^H NMR peaks of primary metabolites identified in baby leaves of *S. oleracea* L.; Figure S1: ^1^H NMR spectra (600 MHz, D_2_O) of eco-sustainable extracts obtained by using ultrasound-assisted extraction (UAE), SLDE-Naviglio (NAV), and maceration (MAC) with EtOH (100), EtOH:H_2_O 80:20 *v*/*v* (80), EtOH:H_2_O 70:30 *v*/*v* (70), and EtOH:H_2_O 50:50 *v*/*v* (50); S1: Samples; S2: LC-ESI/HRMSMS analysis; S3: Multivariate data analysis by LC-ESI/HRMS; S4: Isolation of compounds; S5: NMR analysis; S6: Multivariate data analysis by ^1^H-NMR; S7: Quantitative analysis of 20-hydroxyecdysone (**6**).
