# Supplementary material for: Spinacia oleracea L. Baby Leaves as a Source of Bioactive Principles: The Chemical Profiling of Eco-Sustainable Extracts by Using LC-ESI/HRMS- and 1H NMR-Based Metabolomics
Source: Foods. 2024 Nov 20;13(22):3699. doi: 10.3390/foods13223699 (PMC11593844; doi:10.3390/foods13223699)
Supplement: Supplementary file 1 [file foods-13-03699-s001.zip › foods-3278793-supplementary.pdf]

Supplementary Materials

Spinacia oleracea L. Baby Leaves as a Source of Bioactive Principles: The Chemical Profiling of Eco-Sustainable Extracts by Using LC-ESI/HRMS- and ^{1}H NMR-Based Metabolomics

Antonietta Cerulli [1,2], Luciana Maria Polcaro [1,3], Milena Masullo [1] and Sonia Piacente [1,2,*]

[1] Department of Pharmacy, University of Salerno, Via Giovanni Paolo II 134, 84084 Fisciano, Italy; acerulli@unisa.it (A.C.); lpolcaro@unisa.it (L.M.P.); mmasullo@unisa.it (M.M.)
[2] Agritech National Research Center, Corso Umberto 40, 80138 Naples, Italy
[3] PhD Program in Drug Discovery and Development, Università degli Studi di Salerno, Via Giovanni Paolo II 132, 84084 Fisciano, Italy
* Correspondence: piacente@unisa.it

Table S1. Characteristic ^{1}H NMR peaks of primary metabolites identified in baby leaves of *S. oleracea* L.

Figure S1. ^{1}H NMR Spectra (600 MHz, D_2O) of eco-sustainable extracts obtained by Ultrasound Assisted Extraction (UAE), Naviglio (NAV), and maceration (MAC) with EtOH 100, v/v (100), EtOH: H_2O 80:20 v/v (80), EtOH: H_2O 70:30 v/v (70), EtOH: H_2O 50:50 v/v (50).

S1. Samples

methanol-d_4 (99.95%, Sigma-Aldrich). The peak of TPS (3-(Trimethylsilyl)-propionic-2,2,3,3-d4 acid sodium salt at 0.9% (w/w) in D_2O 99.9 atom%).

S2. LC-ESI/HRMSMS Analysis

S. oleracea extracts were analyzed by liquid chromatography coupled to mass spectrometry with an electrospray ionization source and a linear trap-Orbitrap hybrid analyzer (ThermoFischer, Waltham, MA, USA), operating in negative ionization mode. LC-ESI/HRMS analysis was performed on a Kinetex EVO 5.0 µm column (150 mm × 2.1 mm) (Phenomenex, Aschaffenburg, Germany), using a flow rate of 0.2 mL/min. A binary solvent system was used (eluent A: water with 0.1% formic acid, eluent B: acetonitrile with 0.1% formic acid). The column was held at 30°C during separations and re-equilibration. The following gradient was used: gradient started from 5% B and held at 5% B for 5 min, from 5 to 95% B in 35 min, held at 95% B for 5 min.

The analysis was performed in negative ionization mode and the ESI source parameters are as follows: capillary voltage -48 V; capillary temperature 280 °C; spray voltage 5 kV; tube lens voltage –176.47; gas flow rate in the sheath 15.5%; sweep gas 0. The acquisition was carried out in the range of m/z 130-1600. Data was acquired through both an MS1 scan and MS/MS. Xcalibur version 2.1 software was used to control the instrument for data acquisition and analysis.

S3. Multivariate Data Analysis by LC-ESI/HRMS

Each eco-sustainable extract was acquired in triplicate by LC-ESI/HRMS analysis (negative ion mode). MZMine 2.38 was used for LC-ESI/HRMS data processing (http://mzmine.sourceforge.net (accessed on 13 July 2023)). MZMine software was used to filter the noise and detect and align the peaks observed LC-ESI/HRMS profiles (noise level $5.0x10^3$) successively, cvs file was exported and analyzed for targeted analysis by Simca 12.0 software.

S4. Isolation of Compounds

EtOH: H_2O (70:30, v/v) extract obtained by Naviglio extraction was purified directly by RP-HPLC-UV setting the wavelength at 330 nm. The elution gradient was obtained using water with 0.1% formic acid as eluent A and acetonitrile with 0.1% formic acid as B; the run was carried out with a flow rate of 2.0 mL/ min. A Phenomenex Sinergy Hydro Prep MS C18 column (250 mm × 10 mm, 10 micron) was used. The following HPLC gradient conditions were used: 0 min 5 % B, 5 min 5 % B, 7 min 32 % B, 34 min 32 % B, 36 min 33 % B, 38 min 34 % B, 40 min 35% B, 50 min 50%; 55 min 100%, 60 min 100%. Trigonelline (3.02 mg, t_R = 5.70 min), and **18** (3.6 mg, t_R = 24.51 min), **19** (3.6 mg, t_R = 24.51 min) and **20** (3.00 mg, t_R = 26.55 min).

The same extract was fractionated with Sephadex LH-20 (Pharmacia), fractions 12-16 were purified by HPLC-RI using a Supercosil LC-18 column (250 mm × 10 mm, 5 micron) with 45% MeOH:H_2O hydroalcoholic solution as mobile phase, in this way compound **6** was

isolated (2.2 mg, t_R = 9.0 min); fractions 22-24 were purified in the same condition to isolate tryptophan (1.2 mg, t_R = 7.5 min) and compound **14** (1.0 mg, t_R = 8.0 min).

S5. NMR Analysis

NMR analyses were carried out on a Bruker Ascend-600 spectrometer (Bruker BioSpin GmBH, Rheinstetten, Germany) equipped with a Bruker 5 mm. Methanol-d4 (99.95%, Sigma-Aldrich) was used as solvent for each extract. The NMR data were processed using TopSpin 3.2 software. For HSQC, a spectral width of 12 ppm and 165 ppm in the proton and carbon dimensions, respectively, were used with 1 K data points, 64 scans, 256 t1 increments, and a recycle delay of 2 s. HMBC was obtained with a spectral width of 12 ppm and 230 ppm in the proton and carbon dimensions, respectively, 4 K data points, 120 scans, 256 t1 increments, and a recycle delay of 2 s.

Free Induction Decay (FID) signal data were analysed to Chenomx NMR Suite 10.0 (Chenomx Inc., Edmonton, AB, Canada) software. Data were automatically zero-padded and Fourier transformed (FT). The data were then carefully phased and baseline distortion was corrected. Compounds were identified by matchingthe spectral signals with the 600 MHz librariy from Chenomx. The reference compound TSP was used as an internal standard for the chemical shifts (set to 0 ppm) and as a reference signal for quantification. Quantification of the data was performed by comparing the integration of a known reference signal (TSP) with the signals from a library of compounds containing chemical shifts and peak multiplications for all resonances of the constituents. The results were exported to an Excel file for further analysis. The quantitative results are presented as mean values of the standard deviation (SD) of three indipendent experimets.

S6. Multivariate Data Analysis by ^{1}H-NMR

For the targeted multivariate analysis, all samples were acquired in methanol-d_4 (99.95%, Sigma-Aldrich). The peak of TPS (3-(Trimethylsilyl)-propionic-2,2,3,3-d4 acid sodium salt at 0.9% (w/w) in D_2O 99.9 atom%) at 0 ppm was used as the chemical shift external reference. All samples were run at 300 K, using the zgesgp pulse sequence; the relaxation delay was 4.0 s, and the acquisition time was 5.45 s, with 128 number scans and data collected into 64 k data points. Each free induction decay (FID) was zero-filled to 128 k data points. Before Fourier transformation, an exponential window function with a line broadening factor of 0.3 Hz was applied. The data were processed by Chenomx NMR Suite 10.0 (Chenomx Inc., AB, Canada). Compounds were identified using the Chenomx 600 MHz libraries. The metabolite concentrations were determined using the concentration of a known reference signal (TSP).

*S7. Quantitative Analysis of 20-hydroxyecdisone (**6**)*

Quantitative analyses of compound **6** was performed on a LC-ESI/QTrap/MS/MS system working in Multiple Reaction Monitoring (MRM) mode. HPLC separation was performed by Kinetex Omega 1.6 μm RP C18 column (100 mm 2.1 mm i.d) at a flow rate of 0.3 μL/min. Linear gradient elution was achieved by using H_2O (phase A) and acetonitrile (phase B), both at with 0.1% formic acid (99.9:0.1, v/v). The HPLC gradient started at 5% B; after 2.1 min, % B was at 15%, changing from 15% B to 35% B in 3.3 min, from 35% B to 80%

B in 2.1 min, and returning to the starting percentage in 2.0 min. The instrument operated in the negative ion mode, for the external standard 20-hydroxyecdisone (**6**), following parameters were set: declustering potential -42.4, focusing potential, entrance potential -2.85, collision energy -35.0, and collision cell exit potential -33.7. For the *S. oleracea* eco-sustainable extracts were prepared solutions of 0.5 mg/mL diluted by using methanol and injected in triplicate; solutions of different ES concentrations (0.1, 1.0, 5.0, 10.0, 20.0, 40.0 g/mL) were used. In this way, a calibration curve, analyzed by linear regression (y = 19.1x + 740, R^2 = 0.997) was obtained.

Table S1. Characteristic ^1H NMR peaks of primary metabolites identified in baby leaves of *S. oleracea* L.

**compounds**	**^1H chemical shift (multiplicity, *J* in Hz)**
alanine	1.48 (d, 7.2)
allantoin	6.01 (s)
asparagine	2.95 (dd, 16.9, 7.3)
aspartic acid	2.85 (dd, 16.0, 3.0)
betaine	3.90 (s)
folic acid	7.55 (d, 8.27)
fructose	4.01 (d, 2.3)
GABA	1.90 (m)
glucose	4.62 (d, 8.0)
glutamine	2.53 (m)
3-hydroxyisobutyric acid	1.12 (d, 6.0)
homocysteine	2.24 (m)
isoleucine	0.91 (d, 7.0)
leucine	1.73 (m)
malic acid	4.29 (d, 6.0)
phenylalanine	7.32 (d, 80)
protocatechuic acid	7.41 (d, 2.2)
succinic acid	2.43 (s)
sucrose	5.38 (d, 4.0)
threonine	1.32 (d, 6.6)
trigonelline	9.11 (s)
tryptophan	7.54 (d, 8.1)
uridine	7.91 (d, 7.0)
valine	1.00 (d, 6.8)

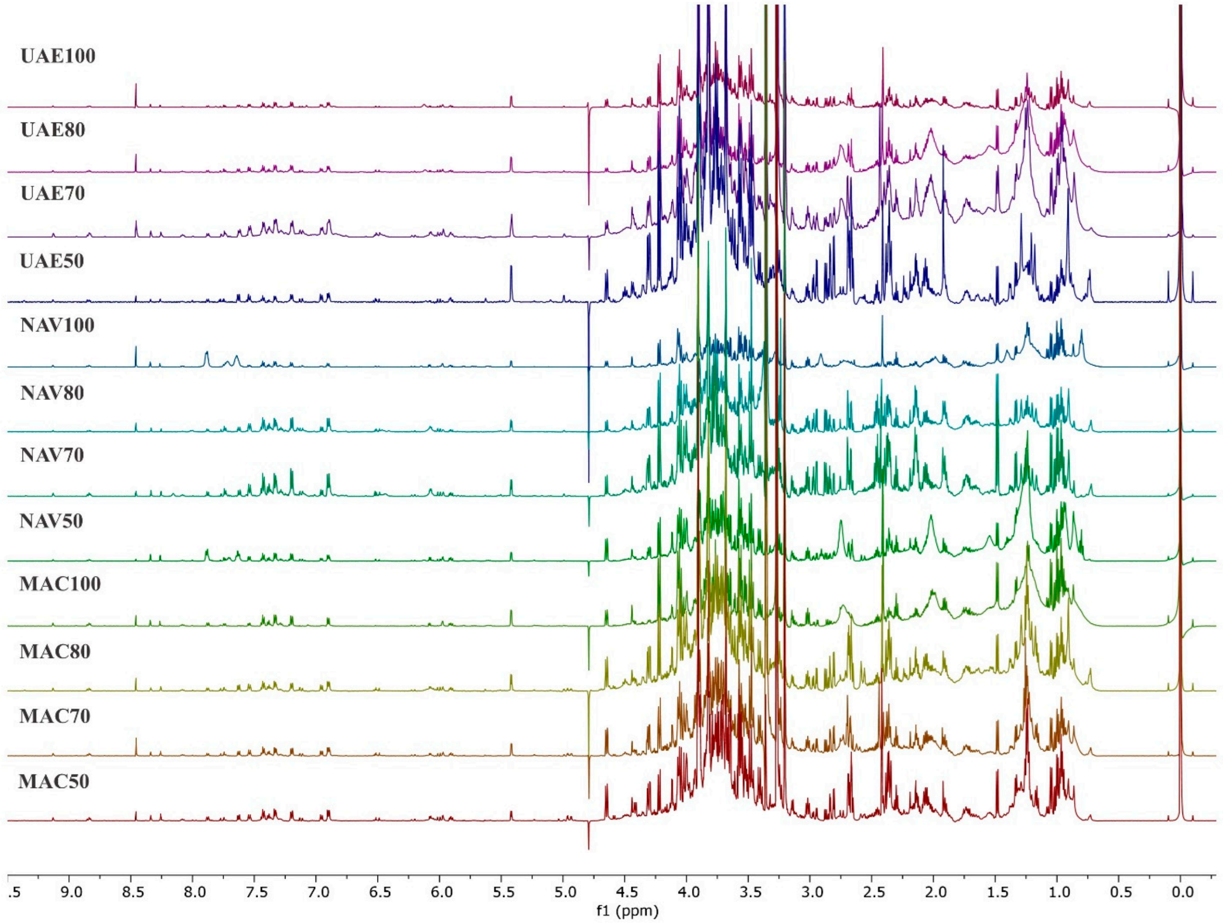

Figure S1. ^{1}H NMR Spectra (600 MHz, D_2O) of eco-sustainable extracts obtained by Ultrasound Assisted Extraction (UAE), Naviglio (NAV), and maceration (MAC) with EtOH 100, v/v (100), EtOH: H_2O 80:20 v/v (80), EtOH: H_2O 70:30 v/v (70), EtOH: H_2O 50:50 v/v (50).
